# Quercetin promotes in vitro maturation of oocytes from humans and aged mice

**DOI:** 10.1038/s41419-020-03183-5

**Published:** 2020-11-11

**Authors:** Yongzhi Cao, Haibin Zhao, Zhao Wang, Changming Zhang, Yuehong Bian, Xin Liu, Chuanxin Zhang, Xin Zhang, Yueran Zhao

**Affiliations:** 1 Center for Reproductive Medicine, Cheeloo College of Medicine, Shandong University, 250012 Jinan, Shandong China; 2 National Research Center for Assisted Reproductive Technology and Reproductive Genetics, Shandong University, 250012 Jinan, Shandong China; 3 Key laboratory of Reproductive Endocrinology of Ministry of Education, Shandong University, 250012 Jinan, Shandong China; 4 Shandong Provincial Clinical Medicine Research Center for Reproductive Health, Shandong University, 250012 Jinan, Shandong China; 5 Central Laboratory, Shandong Provincial Hospital, Cheeloo College of Medicine, Shandong University, 250021 Jinan, Shandong China

**Keywords:** Senescence, Ageing

## Abstract

Maternal fertility declines irreversibly with aging, and advanced maternal age is mostly related to impaired oocyte quality. The flavonol compound quercetin is considered to be an anti-aging agent due to its cytoprotective actions as an antioxidant. However, its role and mechanisms on aged oocytes are unclear. In this study, the quercetin promotes in vitro maturation (IVM) and early embryonic development of oocytes from aged mice. It is extended these findings in human oocytes, showing that quercetin promotes the IVM rate by 19.6% and increases the blastocyst formation rate by 15.5% compared to untreated controls. The overall oocyte quality of aged mice is improved by quercetin treatment, assessed as spindle/chromosome morphology and cortical granule distribution. Mitochondria is the primary endogenous source of age-related oxidative stress, and an RNA-seq analysis of quercetin-treated oocytes reveals molecular insights including scavenged mitochondrial-ROS, reduced apoptosis, and improved autophagy. Further, this study demonstrates that quercetin reduces ROS via SIRT3-mediated acetylation of SOD2’s K68 residue. Thus, beyond demonstrating that quercetin confers beneficial mitochondria-related impacts in aged oocytes, this study illustrates a potential strategy to prevent or delay oocyte aging and to improve success rates of assisted human reproductive technologies (ART).

## Introduction

The progressive loss of ovarian follicles accelerates with aging, especially in humans over the age of 38^1^. However, many women worldwide postpone childbearing and therefore potentially face infertility^2^. Decreasing numbers of oocytes, coinciding with declining oocyte quality, together affect gradual decreases in overall ovarian function, and eventually leading to final natural sterility^3^. Moreover, maternal age-related decline in fecundity is associated with increased aneuploidy and poor oocyte maturation, which can lead to detrimental clinical outcomes, for example, congenital anomalies and miscarriage^4^. One major known cause of oocyte aging is increased oxidative damage by excessive reactive oxygen species (ROS), which can result in part from weakened anti-oxidative defense systems^5^. During in vitro maturation (IVM), ROS can be produced in the oocyte with both oxygen sources from the extrinsic medium and intrinsic cellular metabolism, which was originated from the mitochondria^6^. Regardless of the source, ROS can cause DNA damage, cellular dysfunction, and apoptosis^7^. Thus, it is plausible that scavenging mitochondrial oxidative stress in oocytes could help alleviate age-related oocyte aging and fertility decline.

IVM is now a highly impactful procedure used to help retrieve immature oocytes. It is widely used in both scientific research with laboratory animals and human ART, and also notable because it facilitates the development of totipotent embryos^8–10^. By 2015, estimates suggested that more than 5000 babies had been born following IVM^11^, and the procedure has become ever-more-widespread in the ensuing years. Importantly in the clinic, IVM can eliminate the risk of ovarian hyperstimulation syndrome, as it involves only minimal or no prior FSH stimulation, no hCG stimulation, and offers substantial cost reductions for patients receiving often prohibitively expensive fertility treatments^12^. However, compared to conventional in vitro fertilization (IVF), IVM results in substantially lowered success rate and reduced oocyte developmental potentiality, and yet further IVM performance decreases have been associated with increased maternal age^13^.

To reduce age-related oxidative stress and potentially relieve ROS-induced damage, biologists and medical scientists have explored the use of many antioxidant supplementations, including for example resveratrol, growth hormone, melatonin, rapamycin, and coenzyme Q10^13–17^. Quercetin (3, 3′, 4′, 5, 7-pentahydroxylflavone) is a flavonoid natural product compound found in plants such as berries, broccoli, apples, and onions^18^. Previous studies have reported that quercetin can prevent mitochondrial dysfunction by removing oxidation products, scavenging free radicals, and stimulating antioxidant enzymes^19^. Further, studies have demonstrated that quercetin activating antioxidant capacity can alleviate senescence in mice and can extend lifespan in *C. elegans* by 15%^20,21^. Other work has shown that quercetin can enhance the antioxidant capacity of the ovary in menopausal rats, can delay mouse oocyte postovulatory aging^22,23^, and can substantially improve oocyte IVM rates in swine and goat^8,24^. Aging is a nonmodifiable risk and a common factor for suffering from infertility^25^. Hence, the feasibility of using quercetin to delay ovarian aging and promote fertility by IVM should be assessed.

The result shows that quercetin promotes IVM and subsequent formation of blastocysts using aged mice models, and extended these findings with verification of the observed quercetin effects in human oocytes. These studies also confer a protective mechanism against age-related mitochondrial oxidative stress by reduced apoptosis, improved autophagy, and discover quercetin can scavenge oxidative stress via the SIRT3-dependent deacetylation of SOD2 pathway.

## Materials and methods

The protocol for this study was reviewed and approved by the Institutional Review Board of Reproductive Medicine, Shandong University ([2018] Ethical Review #41). All patients in this study have written their informed consent by the Declaration of Helsinki. Unless otherwise noted, all chemicals and reagents used were purchased from the Sigma Chemical Company.

### Oocyte and embryo collection

Kun Ming (KM) female mice that were 9- to 10-months old (when fertility declines rapidly) were used as a naturally aged mice model, and 6- to 8-week-old young female mice were also used in this experiment. House in a temperature-controlled room with 12D:12L (dark vs. light), water and food were available ad libitum. To obtain fully grown GV oocytes, mice were injected intraperitoneally with 10 IU pregnant mare serum gonadotropin (PMSG) (Ningbo Hormone Product Company, China), and cumulus-oocyte complexes were collected by manual rupturing of antral ovarian follicles after 46–48 h. To obtain fully grown GV oocytes, cumulus cells were removed by repeatedly pipetting. Then, oocytes cultured in M16 medium (Sigma, USA) and covered with mineral oil were incubated under 6% CO_2_, 5% O_2_, and 90% N_2_ at 37 °C for 4 h or 16 h to determine the GVBD ratio or PB1 extrusion rate, respectively. To induce superovulation, intraperitoneal injection of 10 IU PMSG was followed 48 h later by 10 IU human chorionic gonadotropin (hCG) (Ningbo Hormone Product Company, China). After 16 h, superovulated mice were killed, and oviductal ampullae were broken to release cumulus-oocyte complexes, which were subsequently fertilized with adult KM male sperm from the cauda epididymidis. Zygotes in small drops of G-IVF medium (Vitrolife, Sweden) and covered with mineral oil were incubated under 6% CO_2_, 5% O_2_, and 90% N_2_ at 37 °C to observe embryonic developmental potential.

### Oocyte/embryo treatment and microinjection

For the IVM experiment, GV oocytes were washed at least three times and immediately cultured in M16 medium supplemented with or without different concentrations of quercetin. Oocyte maturation rates were observed after 16 h. Quercetin (Q4951, Sigma, USA) was dissolved in DMSO and diluted to a final concentration of 5 μM, 10 μM, or 20 μM. The final concentration of DMSO was less than 0.05%. For the IVF experiment, the embryos were cultured in G1-IVF and G2-IVF media supplemented with or without 10 μM quercetin. Fertilized oocytes developed to the 2-cell stage after 12 h and to the blastocyst stage after 4.5 days. Embryonic development and morphology were examined with a stereomicroscope (Nikon SMZ1500). For microinjection in knockdown experiments, about 10 pL *Sirt3*-targeting siRNA (10 ng/μL) was injected into GV stage oocytes, which were arrested at the GV stage in M16 medium containing 2.5 mM milrinone for 20 h to allow synthesis of new protein. For IVM, si*Sirt3* oocytes were cultured in the M16 medium with (si*Sirt3* + quercetin) or without (*siSirt3*) 10 μM quercetin. As a control, the same amount of RNase-free-PBS was injected into oocytes. siRNA was obtained from RiboBio, and the sequences are shown in supplementary material Table S3.

### Culturing human oocytes/embryos and quercetin treatment

Human GV /GVBD oocytes from discarded IVF cycles, collected from 57 patients whose ages varied from 22 to 42 years, were randomly divided into two groups for treatment with or without 10 μM quercetin under 6% CO_2_, 5% O_2_, and 90% N_2_ at 37 °C. The demographics and baseline profiles of women with and without quercetin-treated oocytes are shown in supplementary material Table S1. They were cultured for 24 h in IVM medium, which was Medium 199 (Gibco/Life Technologies, USA) supplemented with 0.29 mmol/L sodium pyruvate (Sigma, USA), 10% human serum albumin (Vitrolife, Sweden), 0.075 IU/mL recombinant follicle-stimulating hormone (FSH, Merck Serono, Switzerland), 0.15 IU/mL hCG, and 10 ng/mL epidermal growth factor (EGF, Sigma, USA). M II stage oocytes were counted, and the criterion for nuclear maturation was the extrusion of the PB1. After maturation, M II oocytes were used for the ICSI protocol, carried out under an inverted microscope (Nikon, Japan). Zygotes were cultured sequentially with G1-IVF and G2-IVF culture media (Vitrolife, Sweden) and incubated under 6% CO_2_, 5% O_2_, and 90% N_2_ at 37 °C. Embryo photomicrographs were taken at different stages of development. The fertilization rate was calculated 16–18 h after ICSI. The blastocyst rate was evaluated 5–6 days after fertilization, and the blastocyst stage was assessed according to Gardner’s criteria.

### ROS assessment

For measurements of intra-oocyte reactive ROS, oocytes with or without 10 μM quercetin were collected and subsequently incubated for 30 min at 37 °C in M2 supplemented with 10 mM carboxy-H2DCF diacetate (Beyotime, China). After being washed in M2 medium at least 3 times, oocytes were imaged with a confocal microscope (Dragonfly, Andor Technology, UK), and identical settings and 488 nm excitation used in all observations. The fluorescence intensity for each oocyte was measured with Image J (National Institutes of Health, USA).

### Measurement of cytoplasmic ATP content in oocytes

ATP content measurements were made using an EnSpire Multimode Plate Reader (PerkinElmer, USA) and a commercial assay kit based on the luciferin–luciferase reaction (Bioluminescent Somatic Cell Assay Kit, Sigma, USA). Mixed for 5 s before detection, a formula derived from linear regression analysis of a standard curve containing 11 ATP concentrations ranging from 10 fmol to 10 pmol was used to calculate oocyte ATP content. At least 60 oocytes were used for each experiment.

### Immunofluorescence microscopy

In brief, oocytes were fixed for 30 min with 3.7% paraformaldehyde in PBS, treated with 0.5% protease in M2 for 10 s to remove zona pellucida, permeabilized at 37 °C for 10 min with 0.1% Triton X-100 in PBS, blocked with 3% BSA in PBS at 37.5 °C for 0.5 h, and blocked with 1% BSA dissolved in PBS. Oocytes were incubated with primary antibodies in 1% BSA overnight at 4 °C for immunofluorescence staining. The primary antibodies were as follows: anti-LC3 (Cell Signaling, USA), anti-SOD2K68ac (Abcam, USA), and anti-active caspase-3 (Abcam, USA). After being washed three times for 5 min in PBS containing 1% Tween 20 and 0.01% Triton X-100, oocytes were incubated with secondary antibody (Cell Signaling, USA) at room temperature for 1 h. For tubulin staining, oocytes were probed with an anti-α-tubulin antibody (Sigma, USA). To evaluate the distribution of cortical granules, oocytes were probed with labeled lens culinaris agglutinin (Vectorlabs, USA). For the TUNEL method, an in situ cell death kit (Roche, Germany) was used. To detect mitochondrial membrane potential (MMP), oocytes were incubated in M2 medium containing 2 μM JC-1 (Invitrogen, USA) for 30 min at 37 °C. To evaluate mitochondrial distribution, oocytes were cultured in M2 medium containing 200 nM MitoTracker-Red (Invitrogen, USA) for 30 min at 37 °C. To visualize chromosomes, oocytes were counterstained with DAPI (Solarbio, China) for 10 min. Immunofluorescence analysis was always performed using control and quercetin-treated oocytes manipulated in parallel and under identical conditions. After being washed three times, oocytes were mounted on medium (Vector, USA) and examined under a laser scanning confocal microscope (Dragonfly, Andor Technology, UK). The mean fluorescence intensity of each oocyte was measured with Image J (National Institutes of Health, USA). Images were acquired by using the same confocal microscope settings within and between experiments. The antibodies used in these experiments are shown in supplementary material Table S2.

### RNA sequencing and qPCR

For RNA sequencing, 15 M II stage oocytes after IVM from three mice were considered one group, and three replicates were assessed per group. Samples included groups collected with or without 10 μM quercetin treatment. The RNA sequencing protocol, briefly, the sample library was built with a smart-seq HT Kit (Takara, Japan) at Shanghai Sinomics Corporation and sequenced with an Illumina NovaSeq 6000 (Illumina, USA). RNA-sequencing reads were aligned to the GRCm38.91 reference genome using Hisat2 software. Raw data files are publicly available from the Gene Expression Omnibus (GEO) database under accession number GSE157212. Total RNA was extracted from samples using the RNeasy Mini Kit (Qiagen, Germany) by the manufacturer’s protocol. cDNA was obtained by reverse transcription of RNA by PrimeScript reverse transcriptase (Takara, Japan). Expression levels of mRNAs were partially verified by quantitative real-time PCR experiments performed with a Light Cycler 480 (Roche, Germany). The mRNA levels were normalized to endogenous GAPDH mRNA levels (internal control) using calculations performed with Microsoft Excel. The primers used were designed by Primer Premier 5.0 software. Primer sequences are shown in supplementary material Table S3.

### Electron microscopy

To compare the effects of control and 10 μM quercetin treatment, 10 oocytes from five mice were used for each sample. To analyze mitochondrial ultrastructure, oocytes were prepared for transmission electron microscopy (TEM). Briefly, oocytes were cut into 70 nm ultrathin sections, stained with uranyl acetate and lead citrate, and then examined by TEM (Jeol, Japan). Morphometry of mitochondrial ultrastructure was determined from electron micrographs at 30,000-fold magnification. To quantify abnormal mitochondria, ten different fields of each oocyte were counted in a blinded fashion.

### Western blot analysis

For total protein extraction, 100 oocytes for each group were lysed by boiling in SDS buffer for 5 min. After electrophoresis with 12% SDS-PAGE gels, the samples were transferred to a PVDF membrane (Millipore, USA), blocked with 5% skim milk diluted in PBST for 1 h at room temperature, incubated with primary antibody overnight at 4 °C, and incubated with goat anti-rabbit IRDye 680RD (LI-COR Bioscience, USA) or goat anti-mouse IRDye 800CW (LI-COR Bioscience, USA) for 1 h at room temperature. Immunoreactive bands and molecular weight were detected using the Odyssey Infrared Imaging System (LI-COR Bioscience, USA). The antibodies used in these experiments are shown in Supplementary material Table S2.

### Statistical analyses

All experiments were replicated more than three times, and the data obtained were subjected to statistical analysis. Data are presented as mean ± SD unless otherwise indicated. Differences between two groups were analyzed for statistical significance using two-tailed unpaired Student’s t-tests. Multiple comparisons between more than two groups were analyzed by one-way ANOVA test using Prism 5.0. *P* < 0.05 was considered to be significant.

## Results

### Treating oocytes from aged mice with quercetin improves oocyte maturation and early embryonic development

**Fig. 1:**
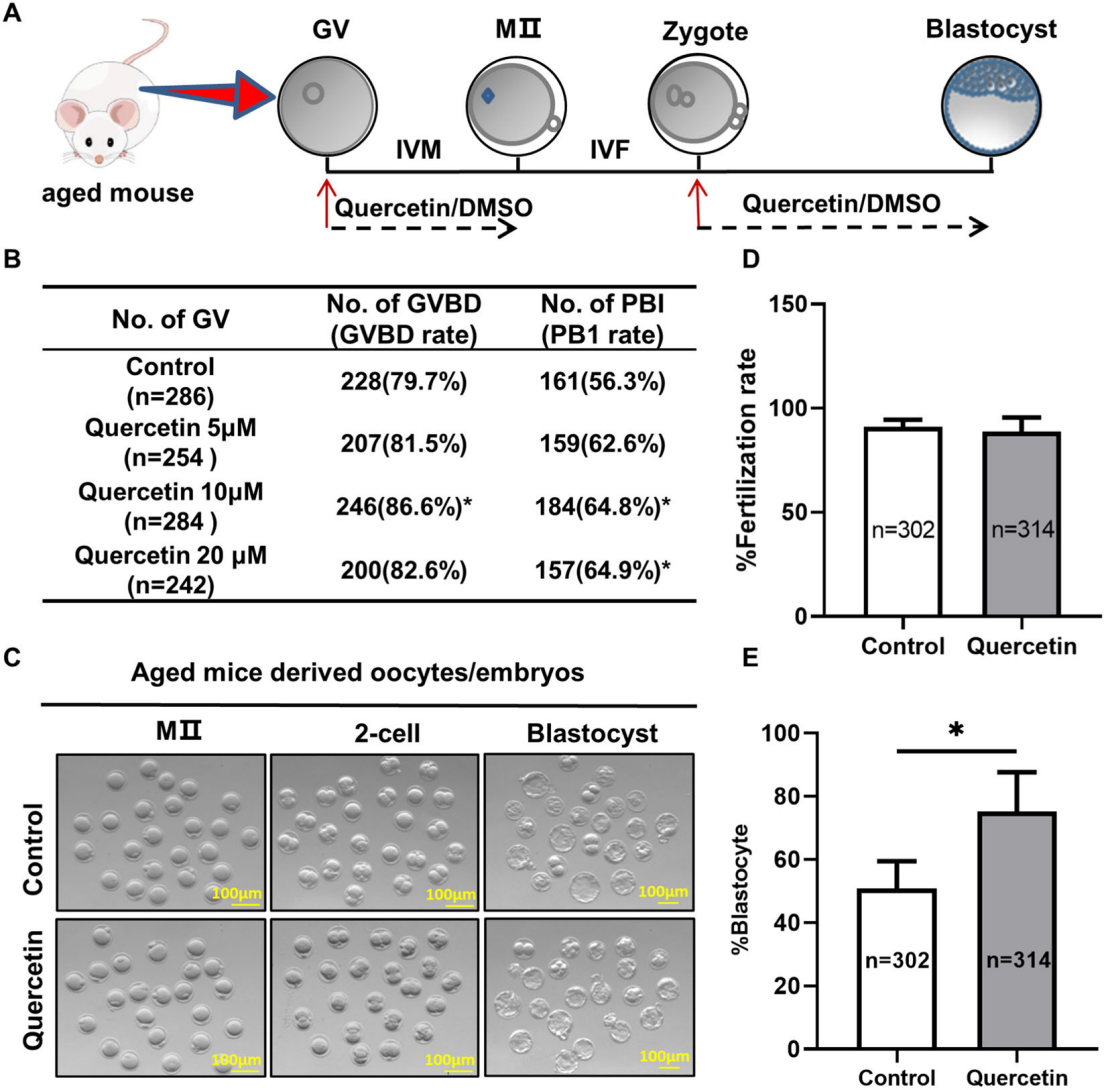
Treating oocytes from aged mice with quercetin improves oocyte maturation and early embryonic development. **A** Timeline of quercetin-treated oocyte development and early embryonic development up to the blastocyst stage. **B** Quantitative analysis of GVBD and Pb1 extrusion in the presence of 0, 5, 10, and 20 μM quercetin. **C** Representative images of oocytes and embryos cultured in vitro at the MII, 2-cell, and blastocyst stages. Scale bar, 100 μm. **D** Quantitative analysis of fertility rate with or without 10 μM quercetin treatment. (**E**) Quantitative analysis of blastocyst formation rate with or without 10 μM quercetin treatment. Data are expressed as means ± SD from three independent experiments. *P < 0.05 vs. control as calculated by two-tailed unpaired Student’s *t*-tests.

To investigate the effect of quercetin on oocytes from aged mice, we performed an IVM test supplemented with different quercetin concentrations (Fig. 1A). A total of 1,066 GV-stage oocytes from 9- to 10-month-old female mice were collected and cultured in M16 medium supplemented with 0 μM, 5 μM, 10 μM, or 20 μM quercetin for 16 h. The 10 μM quercetin treatment surpassed controls and outperformed the other treatment concentrations for both the germinal vesicle breakdown (GVBD) rate and the first polar body (Pb1) extrusion rate (Fig. 1B). Hence, it is selected 10 μM quercetin for further study. These results suggest the treating oocytes from aged mice with 10 μM quercetin can significantly promote the IVM rate.

To examine the potential impacts of quercetin on early embryonic development, IVF in aged mice was performed (Fig. 1A). In culture medium supplemented with 10 μM quercetin or control medium, MII stage oocytes were fertilized with sperm from adult males with normal fertility and spermatogenesis. It is no difference in the rate of 2-cell stage oocytes between the two groups (Fig. 1D). However, the blastocyst formation rate of the quercetin-treated group was significantly increased by 24% than the untreated group (Fig. 1E). Consistent with these findings, our results demonstrated that quercetin had the potential to improve IVM and embryonic developmental competency in aged mice (Fig. 1C).

### Quercetin improves oocyte quality from aged mice

**Fig. 2:**
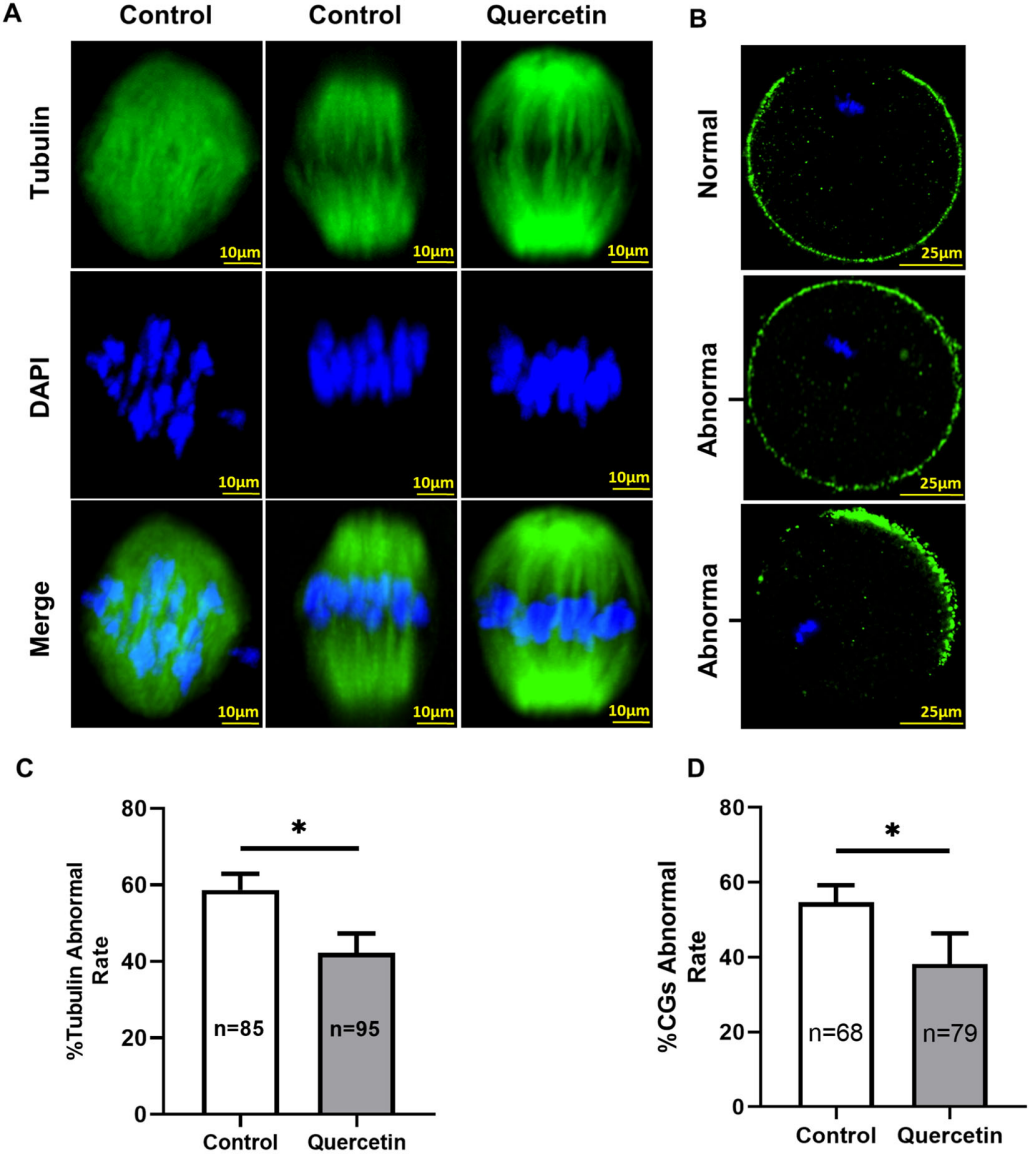
Quercetin improves oocyte quality in aged mice. **A** Representative images of spindle/chromosome organization in oocytes from aged mice with or without 10 μM quercetin treatment. Spindles were stained with α‐tubulin antibody (green), and chromosomes were counterstained with DAPI (blue). Scale bar, 10 μm. **B** Distinct cortical granules (CGs) distributions with or without quercetin treatment. CGs in oocytes were stained with lens culinaris (LCA)‐FITC (fluorescein isothiocyanate: green), and chromosomes were stained with DAPI (blue). Scale bar, 25 μm. **C** Quantification of abnormal spindle/chromosomes oocytes with or without quercetin treatment. **D** The proportion of oocytes with abnormally distributed CGs, following quercetin treatment. Data are means ± SD of at least three independent experiments. *P < 0.05 vs. control as calculated by two-tailed unpaired Student’s *t*-tests.

According to previous studies, reduced oocyte quality (cytoplasmic and nuclear) is one of the major factors causing infertility in aged humans^13^. Abnormal spindle and aneuploidy cause decreased fertilization chances, increased risks for miscarriage, and a rise in progeny with birth defects^17^. To evaluate oocyte quality, firstly comparing spindle morphology and chromosome alignment of M II stage oocytes from aged mice with or without quercetin, the results found various severely abnormal formations in control oocytes, including elongated spindles, no apparent poles, and chromosome misalignment (Fig. 2A). Compared with the control, quercetin treatment significantly decreased the proportion of abnormal spindles and chromosomes (Fig. 2C), as observing bipolar spindles and orderly aligned chromosomes on the equatorial plate.

Because the distributions of cortical granules (CGs) are an important cytoplasmic indicator of the oocyte, they can block polyspermy^26^. To investigate the distribution of CGs in aged oocytes with or without quercetin, oocytes were probed with labeled lens culinaris agglutinin. The results showed that quercetin-treated oocytes led to a significantly higher percentage of normal CGs distributions, which are indicated by a layer of CGs tidily aligned beneath the oolemma and a CG-free domain near the spindle (Fig. 2B). However, over 50% of the control oocytes showed abnormal CGs distribution in aged mice, including increased migration towards the oocyte spindle or oocyte cortex without leaving CG-free domains (Fig. 2D). Taken together, these data imply that quercetin is a powerful agent for combatting age-related quality declines in IVM oocytes.

### Whole transcriptome analysis of quercetin-treated oocytes from aged mice

**Fig. 3:**
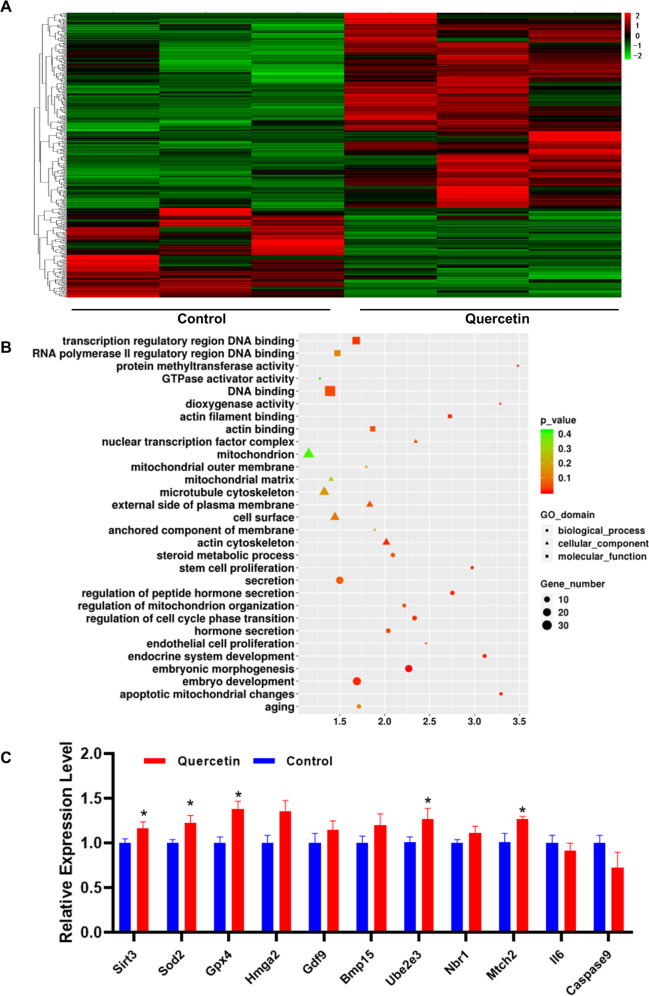
Whole transcriptome analysis of quercetin-treated oocytes from aged mice. **A** Heatmap of mRNA expression profiles in oocytes, showing changes in a subset of genes in response to quercetin treatment. **B** Gene ontology (GO) analysis of the differentially expressed genes after quercetin treatment. **C** Validation of RNA-seq data by qPCR. Data are expressed as means ± SD from three independent experiments. *P < 0.05 vs. control as calculated by two-tailed unpaired Student’s *t*-tests.

To investigate variations in gene expression of quercetin-treated aged mice oocytes after IVM, we performed single-cell RNA sequencing of M II stage oocytes. By comparing the transcriptomes of treated and control oocytes, 124 up-regulated and 270 down-regulated genes were identified cutoff criteria: adjusted P-values less than 0.05 and fold change greater than 2 (Fig. 3A). By using a qPCR analysis of the expression of selected genes, similar results confirmed expression trends from the RNA-seq data (Supplementary Material Fig. S1). Gene Ontology analysis revealed quercetin induces alteration of gene expression related to multiple biological processes, cellular components, and molecular functions, including clear enrichment for metabolic activity and aging terms (Fig. 3B). Numerous up-regulated genes have annotated functions related to anti-oxidative metabolism (*Sod2, Gpx4*), oocyte and embryonic development (*Hmga2, Ube2e3, Bmp15, Gdf9*), autophagy (*Nbr1*), aging (*Sirt3*), and the mitochondrial respiratory chain (*Mtch2*). Some of the down-regulated genes had annotated functions concerning apoptosis (*Il6, Caspase9*) (Fig. 3C). Thus, the transcriptome analysis of oocytes from aged mice identified many genes altered by quercetin treatment and suggested that such treatment has a pronounced impact on genetic programs associated with mitochondrial dysfunction.

### Quercetin improves mitochondrial function in oocytes from aged mice

The decline of oocyte quality is related to mitochondrial dysfunction, which is responsible for molecular and cellular failures of aged oocytes and infertility^27^. Thus, it is hypothesized that culture medium supplemented with quercetin would improve age-induced mitochondrial dysfunction. Therefore, the effect of quercetin on mitochondrial function was assessed in four ways: distribution of mitochondria, mitochondrial membrane potential (MMP), ATP level, and mitochondrial ultrastructure.

**Fig. 4:**
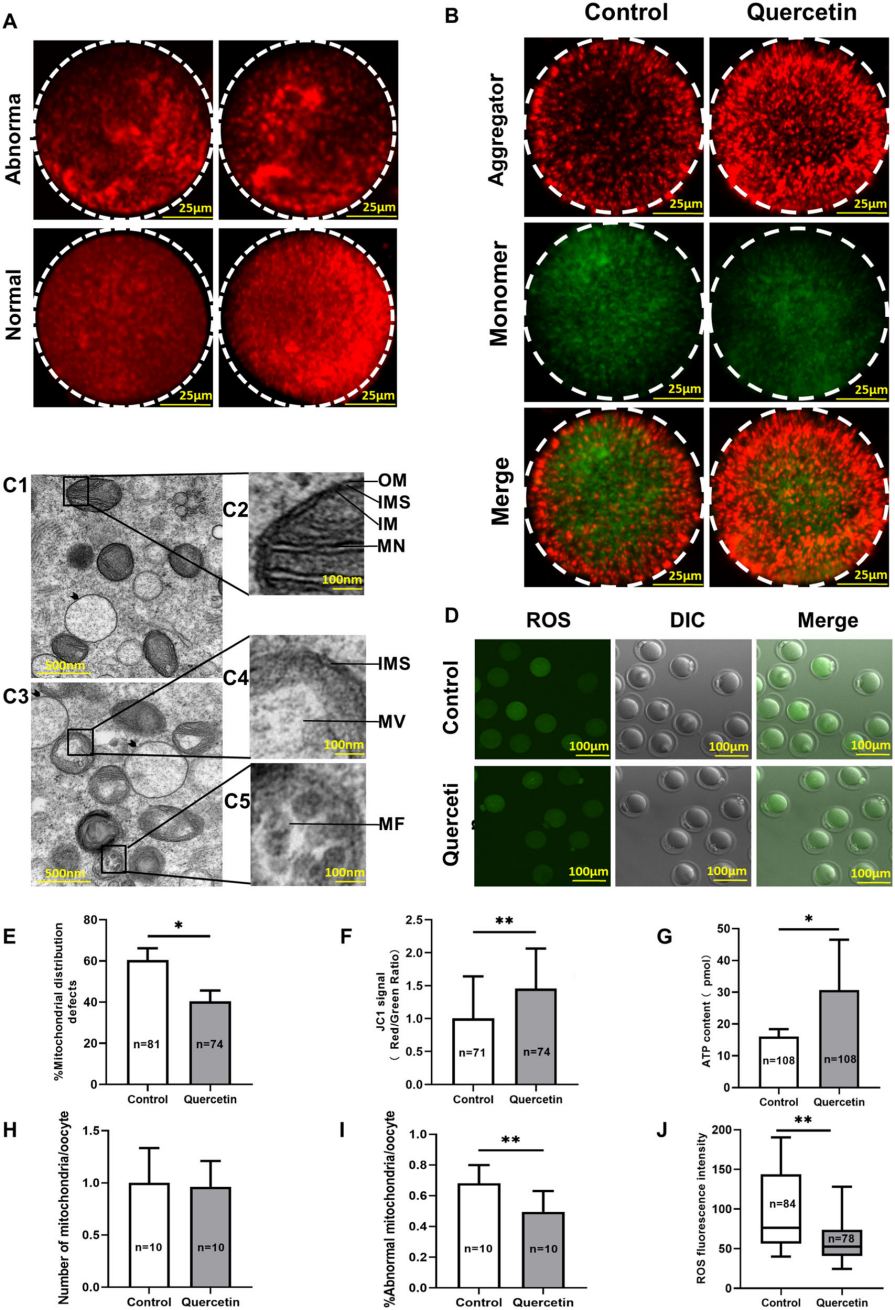
Quercetin attenuated mitochondrial dysfunction of oocytes from aged mice. **A** Confocal micrographs showing the distribution of mitochondria in oocytes treated with or without quercetin. Mitochondria were stained with MitoTracker-Red. Scale bar, 25 μm. **B** Confocal images showing the mitochondrial membrane potential (MMP), as determined by staining intensity with the MMP-specific probe JC-1, for oocytes treated with or without quercetin. Note that the same oocytes were observed in the TRITC channel (red fluorescence) and the FITC channel (green fluorescence). Scale bar, 25 μm. **C1** Representative electron micrograph of mitochondria from an oocyte from the quercetin treatment group. The arrowhead denotes an oocyte wherein the smooth endoplasmic reticulum (SER) vesicles were markedly swollen. Scale bar, 500 nm. **C2** Higher magnification view of the boxed region from C1, highlighting a vacuolated mitochondrion, showing mitochondrial cristae (MC), the structure of the outer membrane (OM), and inner membrane (IM), as well as a well-defined inter-membrane space (IMS). Scale bar, 100 nm. **C3** Representative electron micrograph of mitochondria of the control group oocytes. Scale bar, 500 nm. **C4**, **C5** Higher magnification views of boxed regions in C3, highlighting abnormal mitochondrial ultrastructural features, including the mitochondrial vacuole (MV), a narrowed inter-membrane space (IMS), and myelin figures (MF). Scale bar, 100 nm. **D** Representative images of ROS fluorescence (green) in control and quercetin-treated oocytes. Scale bar, 100 μm. **E** The proportion of oocytes with abnormally distributed mitochondria from the control and quercetin-treatment groups. **F** Quantification of MMP (red/green fluorescence intensity) as determined by staining with the MMP-specific probe JC-1. **G** Quantification of the intra-oocyte adenosine triphosphate (ATP) content, for oocytes treated with or without quercetin. **H** Quantification of the number of mitochondria in M II stage oocytes after in vitro maturation (IVM) with or without quercetin treatment. **I** Quantification of abnormal mitochondrial ultrastructural features in M II stage oocytes after in vitro maturation (IVM) with or without quercetin treatment. **J** Quantification of ROS fluorescent intensity in control and quercetin-treated oocytes. Each group was 10 oocytes from 5 mice. Data are means ± SD of at least three independent experiments. *P < 0.05 vs. control; **P < 0.01 vs. control as calculated by two-tailed unpaired Student’s *t*-tests.

To determine if quercetin treatment can attenuate the abnormal distribution of mitochondria, MitoTracker-Red staining and confocal microscopy were used to examine M II oocytes after IVM. More than 60% of the control group oocytes displayed abnormal mitochondrial distributions, including perinuclear distribution and cluster distribution, whereas the quercetin group oocytes had significantly fewer abnormalities (Fig. 4A, E). These findings indicated that quercetin can apparently alleviate some of the impaired mitochondrial dynamics known to accompany oocyte aging.

Previous reports indicated that MMP might be compromised in aged human oocytes^28^. To assess mitochondrial membrane potential (MMP), another indicator of mitochondrial function, the MMP index (calculated as the ratio of red/green fluorescence intensity by JC-1 staining) was demonstrated a significantly increased the index in the quercetin group compared with the untreated group (Fig. 4B, F). Dysfunctional mitochondria are known to impair an oocyte’s metabolic capacity, which results in decreased adenosine triphosphate (ATP) content in aged oocytes^17^. Quantitative measurements of ATP levels revealed that quercetin dramatically increased the levels in aged mice oocytes over those measured in controls (Fig. 4G).

Ultrastructural aberration of mitochondria can herald mitochondrial-dependent degradation and apoptosis in somatic cells^29^. To determine if quercetin supplementation impacts the mitochondrial ultrastructure of oocytes, transmission electron microscopy (TEM) was used to examine aged oocytes after IVM. Compared with controls, the quercetin-treated oocytes had no difference in the number of mitochondria (Fig. 4H), but fewer abnormalities in mitochondrial ultrastructure (Fig. 4I), which typically exhibit well-aligned mitochondrial outer and inner membranes, and have a well-defined inter-membrane space (Fig. 4C1, C2). The results showed four types of abnormal ultrastructure: mitochondrial vacuoles, narrowed inter-membrane spaces, loss of cristae, and myelin figures (Fig. 4C3–C5). It is also noted that, compared with control oocytes, the quercetin-treated oocytes had fewer swollen vesicles in ultrastructural images of the smooth endoplasmic reticulum and the electron density in the oocyte mitochondrial matrix was higher (Fig. 4C1, C3). These TEM results indicate that quercetin in IVM can apparently alleviate some of the age-related abnormalities known to occur in the mitochondrial ultrastructure of aged oocytes.

As the production of intracellular ROS is known to affect mitochondrial function^6^, we investigated if quercetin treatment can promote oocyte quality by decreasing ROS levels. Oocytes collected and analyzed following quercetin treatment showed significantly reduced ROS levels compared with the control treatment (Fig. 4D, J).

### Quercetin decreases apoptosis and improved autophagy of aged oocytes

**Fig. 5:**
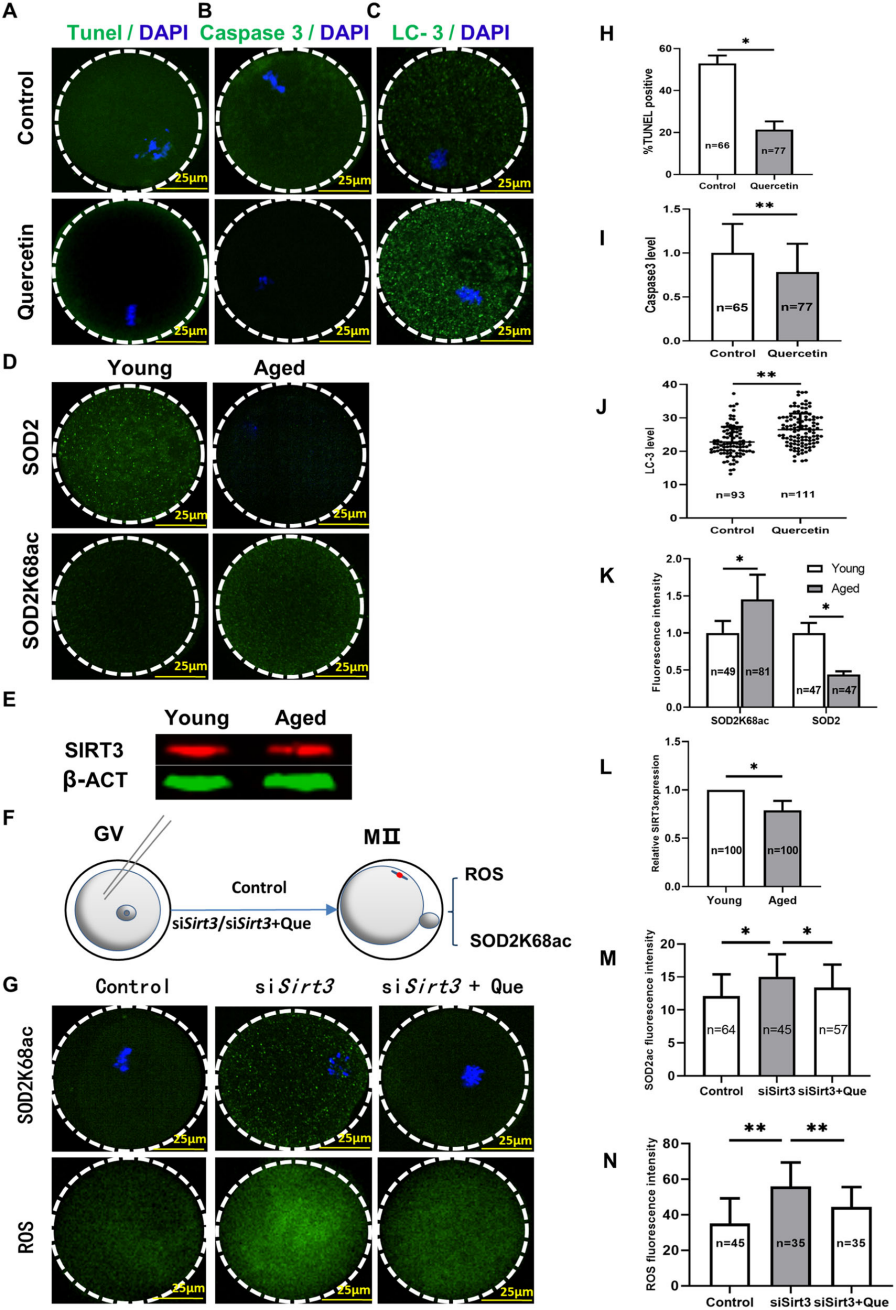
Quercetin decreases apoptosis and increases anti-oxidative activity via SIRT3-mediated reduction of SOD2K68 acetylation in oocytes from aged mice. **A** Confocal images of TUNEL assay examining control and quercetin-treated oocytes. The green fluorescence indicates TUNEL-positive oocytes. Scale bar, 25 μm. **B** Confocal images showing levels of activated Caspase 3 in control and quercetin-treated oocytes. Scale bar, 25 μm. **C** Confocal images showing autophagosomes (LC3-II puncta) in control and quercetin-treated oocytes. Scale bar, 25 μm. **D** Confocal images showing SOD2K68c/SOD2 in young and aged oocytes. Scale bar, 25 μm. **E** Western blot analysis showed reduced SIRT3 expression in GV stage oocytes from aged mice compared with young mice. β-action served as an internal control. **F** Schematic illustration of the experimental protocol to determine whether quercetin treatment reduces ROS accumulation levels and increases the extent of SOD2K68 acetylation by *Sirt3*. **G** Quercetin lowers the acetylation levels of SOD2K68 acetylation and ROS upon the reduction of SIRT3 expression. Scale bar, 25 μm. **H** The percentage of TUNEL-positive oocytes in control and quercetin-treated oocytes. **I** The Caspase3 was determined by immunofluorescence in control and quercetin-treated oocytes. **J** Quantification of LC3 intensity in control and quercetin-treated oocytes. **K** The levels of SOD2K68 are negatively associated with SOD2 enzymatic activity in M II stage oocytes from aged mice and young mice. **L** Western blot analysis showed the reduced Sirt3 expression in oocytes from aged mice compared with young mice. **M** Quantitative analysis of fluorescence intensity showing the acetylation levels of SOD2K68 after injecting Control, siSirt3, and siSirt3 + Quercetin treatment (Que). **N** Quantitative analysis of ROS fluorescence intensity after injecting Control, si*Sirt3*, and si*Sirt3* + Quercetin treatment (Que). DNA was stained with DAPI. Data are means ± SD of at least three independent experiments. Differences between two groups were analyzed by two-tailed unpaired Student’s *t*-tests. Multiple comparisons between more than two groups were analyzed by one-way ANOVA test. *P < 0.05 vs. control; **P < 0.01 vs. control as calculated by two-tailed unpaired Student’s *t*-tests.

ROS triggers mitochondria-mediated oocyte apoptosis in several mammalian species, apoptosis is one of the hallmarks of aging, and previous studies have highlighted an apparently close functional relationship between apoptosis and autophagy in oocytes^30,31^. TUNEL analysis revealed that control oocytes were significantly more likely to exhibit a positive rate compared with quercetin-treated oocytes after IVM (Fig. 5A, H). Furthermore, the finding showed that caspase3 activation was significantly decreased upon quercetin-treated oocytes compared with the untreated group (Fig. 5B, I).

Autophagy is a cell survival mechanism that can help prevent apoptosis by eliminating damaged organelles in lysosomes. Previous work has established that autophagy can improve oocyte mitochondria quality, and LC3 is an autophagy marker protein located on the autophagosome membrane^32,33^. To examine if quercetin affected autophagy in age-related oocytes, confocal images revealed that LC3 was significantly up-regulated by quercetin treatment compared with the control (Fig. 5C, J). Hence, quercetin can apparently help prevent the age-related quality decline of oocytes by reducing apoptosis and improving autophagy.

### Quercetin increases anti-oxidative activity via SIRT3-mediated reduction of SOD2K68 acetylation

Previous reports have suggested that the K68 residue of the SOD2 protein is an acetylation site associated with ROS homeostasis in oocytes^34^. Multiple studies have indicated that SIRT3 may deacetylate SOD2K68, consequently activating SOD2 and thereby potentially increasing its anti-oxidative activity^35^. Based on these findings, we then examined if SOD2 acetylation levels were negatively correlated with its enzymatic activity. Confocal microscopy revealed that oocytes from young mice had significantly lower SOD2K68 acetylation levels, which correspondingly stimulated SOD2 activity compared with that in aged mice (Fig. 5D, K). Moreover, we observed a significant increase in *Sirt3* and *Sod2* expression in the quercetin treatment group relative to their levels in the untreated group (Fig. 3C).

To determine whether SIRT3 expression was altered in GV stage oocytes from aged mice, we conducted protein expression analysis. Western blotting revealed that SIRT3 levels in oocytes of aged mice were significantly reduced compared with that of young mice (Fig. 5E, L). Furthermore, to investigate if the deacetylation state of SOD2K68 (SOD2K68ac) is regulated by SIRT3, fully grown GV oocytes from young mice were injected exogenous si*Sirt3* or PBS, as the negative control. Oocytes were arrested at the GV oocyte stage for about 12 h in the M16 medium supplemented with 2.5 mM milrinone, which interferes with SIRT3 protein synthesis. For IVM, si*Sirt3* oocytes were cultured in the M16 medium with (si*Sirt3* + quercetin) or without (si*Sirt3*) 10 μM quercetin. MII oocytes were collected and labeled with anti-SOD2K68ac antibody and carboxy-H2DCF diacetate to respectively evaluate SOD2K68ac expression and ROS levels (Fig. 5F). Quantitative analysis of confocal microscopy images showed that, compared with control oocytes, the SOD2K68ac level was significantly increased in si*Sirt3* oocytes. In contrast, si*Sirt3* + quercetin dramatically decreased the SOD2K68ac level in oocytes compared with si*Sirt3* oocytes (Fig. 5G, M). Moreover, analysis of ROS levels showed that si*Sirt3* oocytes had significantly elevated ROS levels. The ROS accumulation was still increased in si*Sirt3* + quercetin oocytes compared with control oocytes, but the addition of quercetin caused a significant ROS reduction compared with si*Sirt3* oocytes (Fig. 5G, N).

### Quercetin improves human oocyte in vitro maturation and early embryonic development

**Fig. 6:**
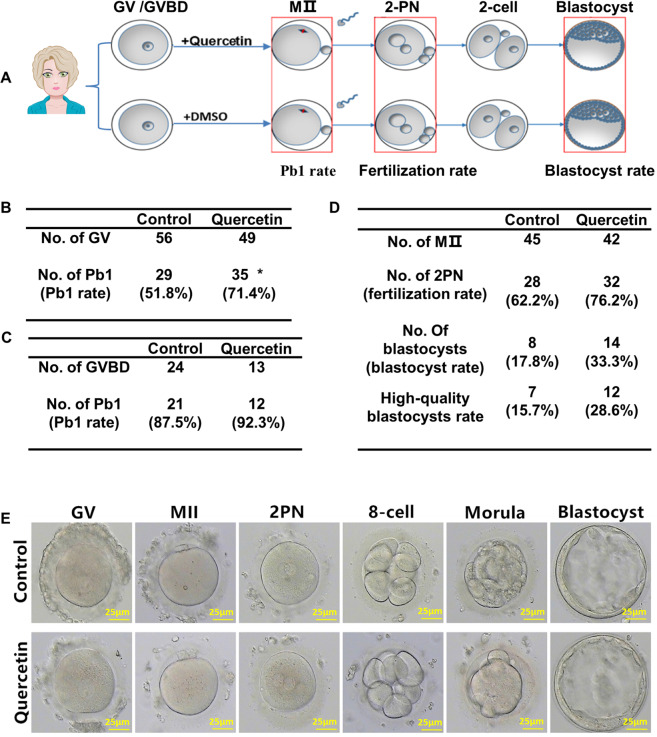
Quercetin improves human oocyte in vitro maturation and early embryonic development. **A** Timeline for the development of quercetin-treated human oocytes and early embryos up to the blastocyst stage. **B** Improved Pb1 extrusion of human embryos after quercetin treatment from GV oocytes. **C** Improved Pb1 extrusion of human embryos after quercetin treatment from GVBD oocytes. **D** Improved fertility rate, blastocyst formation rate, and high-quality blastocysts rate of human embryos after quercetin treatment. **E** Morphology of embryos after in vitro culture with or without quercetin in the culture medium. Scale bar, 25 μm. *P* < 0.05 vs. control as calculated by two-tailed unpaired Student’s *t*-tests.

Having demonstrated that quercetin supplementation of culture medium improved IVM and blastocyst development rates for age-related mice oocytes, it is also examined the effect of 10 μM quercetin on IVM and intracytoplasmic sperm injection (ICSI) stage human oocytes (Fig. 6A, E). For human oocytes IVM, quercetin treatment caused a significant increase from 51.8% to 71.4% (Fig. 6B), and the treating oocytes from GVBD with 10 μM quercetin can also promote the IVM rate (Fig. 6C). Moreover, both the fertilization rate and the blastocyst development rate were much higher under quercetin-treated oocytes. With quercetin treatment, the fertilization rate increased from 62.2% to 76.2%, while the rate of blastocyst formation increasing from 17.8% to 33.3% (Fig. 6D). Moreover, a larger proportion of high-quality blastocysts was observed from cultured embryos treated with quercetin compared with controls (Fig. 6D). Thus, suggesting that quercetin has the potential to improve oocyte maturation and early embryonic development in human ART.

These results suggest that quercetin can help prevent the excessive ROS accumulation that is known to result from decreased *Sirt3* levels in aged oocytes. Moreover, this finding that quercetin up-regulated *Sirt3* expression suggests the presence of a regulatory mechanism through which quercetin can trigger increased cellular anti-oxidative activity, specifically indicating that quercetin acts via a SIRT3-mediated reduction of SOD2K68 acetylation levels (Supplementary Material Fig. S2).

## Discussion

For human ART, IVM of oocytes remains limited by its low success rate, with especially pronounced limitations for aged oocytes. In the present study, these data indicate that quercetin can improve IVM success rates and can ameliorate the quality of aged oocytes, subsequently improving fertilization outcomes. Specifically, these results both confirmed our initial hypothesis about oocyte and fertility-related improvements upon quercetin treatment and provided information about quercetin-related biological mechanisms. Our results confirmed a role for quercetin in preventing the formation of abnormal mitochondrial structures and dysfunctional processes typical of aged oocytes. Specifically detecting that quercetin improves the quality of aged oocytes by reducing the levels of known age-related mitochondrial oxidative stress, reducing the extent of apoptosis, and promoting autophagy. Besides, the finding demonstrated that quercetin may trigger decreased cellular anti-oxidative activity via SIRT3-mediated reduction of SOD2K68 acetylation levels. Finally, our experiments with human oocytes also showed increased IVM success and early embryonic development potential upon quercetin treatment.

It is thought that many effects on embryonic development and clinical outcomes are deeply rooted in the oocyte maturation process^36^. However, because of suboptimal culturing conditions, IVM oocytes have been reported to exhibit embryonic developmental arrest at various stages^37^. So, the addition of effective agents into the IVM culture system is now understood to have great clinical importance. Previous in vitro studies have shown that quercetin can be beneficial for embryonic development potential in swine and goats^8,24^. However, there is to date no direct evidence of any quercetin effects on human oocytes during ART. The present study thus represents the first assessment of quercetin effects on human oocytes. Moreover, our demonstration of multiple quercetin-related improvements in oocytes from aged mice corroborates findings from studies in swine^24,38^. The present study fundamentally supports further exploration of the use of quercetin to improve the IVM of oocytes derived from aging patients suffering from reproductive dysfunction.

Previous studies have shown that at least one in eight women experience infertility^39^. Many factors contribute to female infertility, including for example fallopian tube blockage, endometrial receptivity, and ovulation dysfunction. Nevertheless, age-related fertility decline is understood to be a particularly prevalent reason for infertility, aged women tend to produce fewer oocytes and have lower implantation potential^40^. It bears emphasizing that some work suggests that the reduced quantity of oocytes retrieved from aged maternal may be less important than age-related declines in oocyte quality^41^. A major challenge when seeking to improve the quality of IVM oocytes is the need to synchronize between oocyte nuclear and cytoplasmic maturation^10^. The spindle/chromosome morphology and CGs distribution are known to be important indicators for oocyte nuclear and cytoplasmic maturation, respectively^42^. This study showed that quercetin can attenuate anomalous spindle formation, improve chromosome alignment, help maintain normal CGs distribution, and these results are consistent with a previous report about quercetin effects attenuating morphological changes during postovulatory oocyte aging^23^. Notably, no prior study has evaluated the distribution of CGs in oocytes upon quercetin treatment. Quercetin should be considered as a powerful agent that can combat age-induced quality declines in IVM oocytes. Further, these results also indicate that quercetin could be explored as a potential agent for use in IVM for urgent fertility preservation in for example cancer survivors^43^.

Aging is highly associated with increased senescent cell burden, and some studies have shown that caloric restriction (CR) without malnutrition can prolong maternal fertility^44,45^. However, it has been noted that CR can cause irregular estrous cycles and inhibit oocyte development^16^. Although cryopreservation of gametes and ovarian tissue technologies can help women achieve successful pregnancies^46^, these cannot improve gradual decreases in oocytes quality and ovary function. Quercetin has been previously found to exert diverse fertility-related effects^47^, but physiological mechanisms for these quercetin-related effects remain poorly understood. Emerging evidence indicates that oxidative stress is the most probable factor influencing ovarian aging, and mitochondria are known to be the primary endogenous source of ROS^48^. Mitochondria are also a major site for ROS damage and are the known induction site for the intrinsic induction pathway of autophagy^49^. There are reports that quercetin mainly accumulates in mitochondria of cells^50^. The results strongly suggest that quercetin-treated oocytes reduce oxidative stress levels and reduce the extent of apoptosis. These results therefore support previous studies reporting that quercetin interferes with oxidative stress and apoptosis^51^. More specifically, the study has highlighted an apparently close functional relationship between apoptosis and autophagy in quercetin-treated oocytes. Taken together, this work supports that quercetin can be used as an effective agent to prevent mitochondrial dysfunction and to alleviate the age-related accumulation of excessive ROS levels. These results also suggest that these mitochondrial ROS-related impacts can at least partially explain observed quercetin-treatment-induced improvements in the IVM potential of oocytes derived from aged mice.

The SIRT3 protein is known to mainly localize in mitochondria, where it apparently regulates 80–90% of mitochondrial protein acetylation^52^_._ It is known that *Sirt3* can enhance FoxO3a activity, thereby regulating the activities of SOD2 and CAT, which can positively affect age-induced fertility decline^41^. The results are in agreement with studies, that age-related decline in SIRT3 expression levels^53^. Further, the *Sirt3*-dependent deacetylation of SOD2 promotes ROS accumulation, which is known to contribute to the poor quality of oocytes in diabetic mice^35^. Increased ROS levels are known to stimulate *Sirt3* transcription, leading to SOD2 deacetylation and activation, and yet *Sirt3* can help eliminate ROS by transforming SOD2ac into SOD2^34,54^. This work experimentally the detailed mechanism through which quercetin can trigger increased cellular anti-oxidative activity via *Sirt3*-mediated reduction of SOD2K68ac levels.

Thus, our studies demonstrate that quercetin attenuates age-related mitochondrial oxidative stress and subsequently improves the quality of oocytes, promoting both oocyte maturation and early embryonic development in humans and aged mice oocytes. Considering the known contributions of mitochondrial ROS in reducing oocyte quality, it seems likely that further benefits for preserving oocyte quality could be gained by testing additional anti-mitochondrial-ROS agents. Extending even further, a better understanding of how such agents—and their biological mechanisms of action—directly contribute to improving basic IVM culture systems protocols should help in efforts to develop and improve the presently quite low success rates of this clinically and economically attractive ART tool. Finally, these findings propose a potential strategy for applying quercetin as a measure to help prevent age-related oocyte quality deficits and to improve ART success rates for middle-aged women. However, it bears strong emphasis that any clinical interventions based on quercetin will certainly require rigorous safety evaluations and careful monitoring for unanticipated impacts on pregnancy outcomes.

## Supplementary information

Supplement Figure Legends

Volcano plot showing the downregulated and upregulated genes after quercetin-treated oocytes.

Diagram illustrating the proposed mechanisms for quercetin increasing IVM and blastocyst rates.

Baseline profiles of women with and without quercetin-treated oocytes.

Antibody information

Primer Sequences
