# Supplementary material for: Quercetin promotes in vitro maturation of oocytes from humans and aged mice
Source: Cell Death Dis. 2020 Nov 11;11(11):965. doi: 10.1038/s41419-020-03183-5 (PMC7658351; doi:10.1038/s41419-020-03183-5)
Supplement: Supplementary file 1 — Supplement Figure Legends [file 41419_2020_3183_MOESM1_ESM.docx]

**Fig S1** **Volcano plot showing the downregulated and upregulated genes after quercetin-treated oocytes.** Different dots indicate the transcription change, the red dots indicate upregulated genes, the blue dots indicate downregulated genes, and the gray dots indicate no change in transcription.

**Fig S2 Diagram illustrating the proposed mechanisms for quercetin increasing IVM and blastocyst rates.** Quercetin can attenuate age-related mitochondrial oxidative stress and subsequently improves the quality of oocytes, promoting both oocyte maturation and early embryonic development in human and aged mice oocytes. Quercetin can overcome multiple age-related-decline oocyte phenotypes by reduced apoptosis, improved autophagy, and scavenged oxidative stress via SIRT3-mediated acetylation of SOD2 residue K68.
