# Supplementary figures and images for: Quercetin promotes in vitro maturation of oocytes from humans and aged mice
Source: Cell Death Dis. 2020 Nov 11;11(11):965. doi: 10.1038/s41419-020-03183-5 (PMC7658351; doi:10.1038/s41419-020-03183-5)

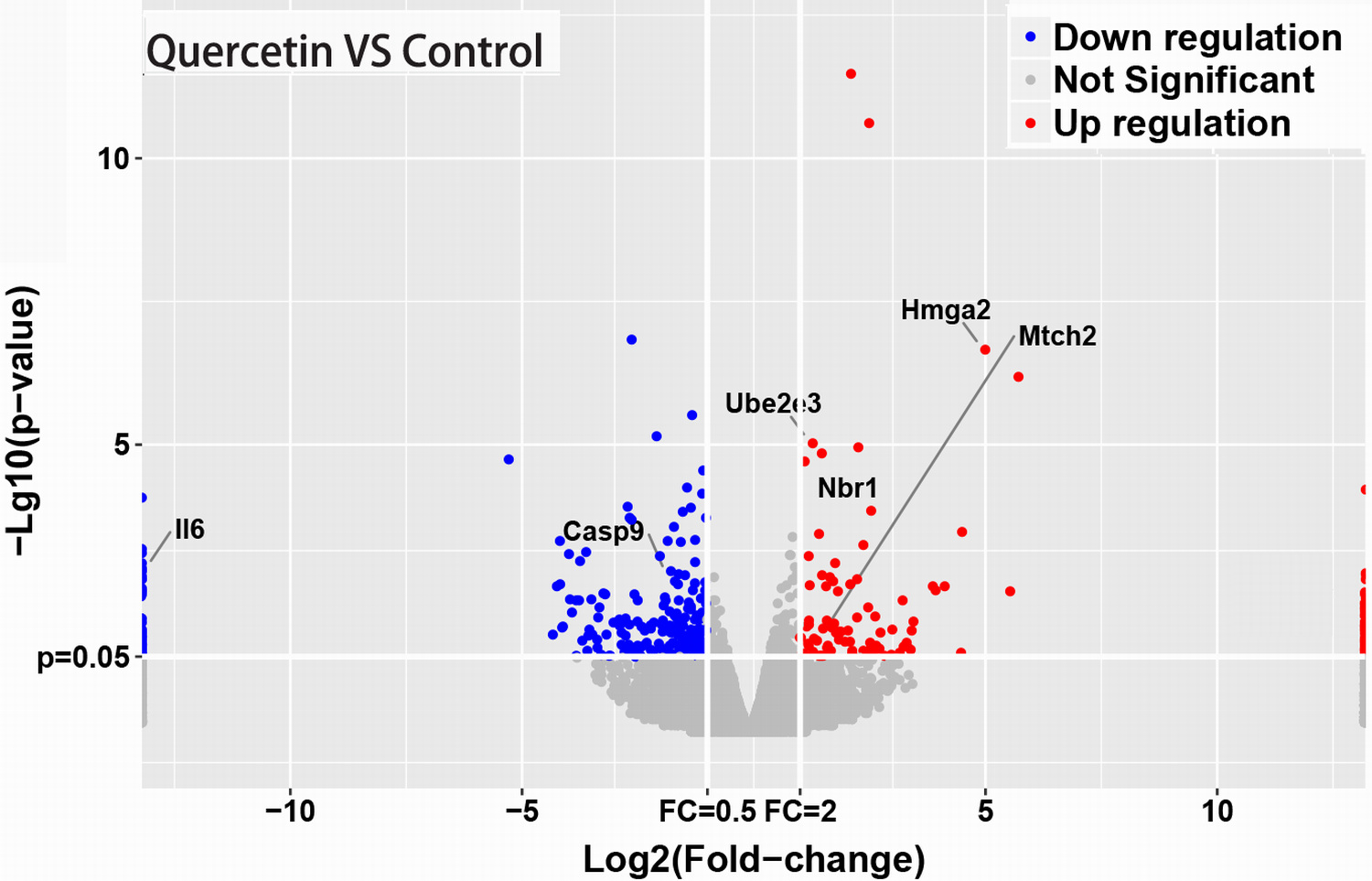

Supplement: Supplementary file 2 — Volcano plot showing the downregulated and upregulated genes after quercetin-treated oocytes. [file 41419_2020_3183_MOESM2_ESM.tif]

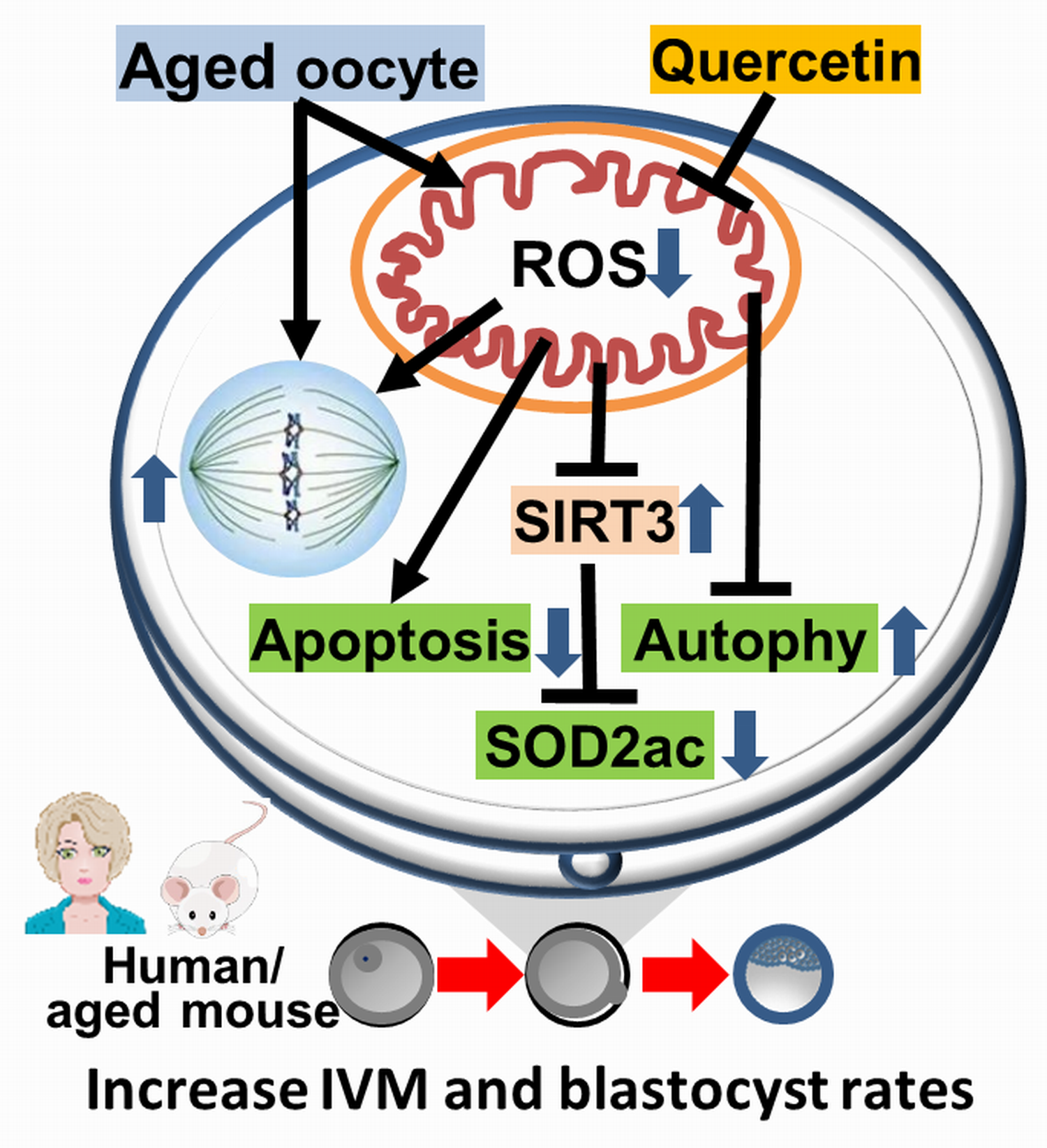

Supplement: Supplementary file 3 — Diagram illustrating the proposed mechanisms for quercetin increasing IVM and blastocyst rates. [file 41419_2020_3183_MOESM3_ESM.tif]
