# Supplementary material for: Quercetin promotes in vitro maturation of oocytes from humans and aged mice
Source: Cell Death Dis. 2020 Nov 11;11(11):965. doi: 10.1038/s41419-020-03183-5 (PMC7658351; doi:10.1038/s41419-020-03183-5)
Supplement: Supplementary file 4 — Baseline profiles of women with and without quercetin-treated oocytes. [file 41419_2020_3183_MOESM4_ESM.docx]

**Table S1. Baseline profiles of women with** **and without quercetin-treated oocytes.**

|  | **Control(n=33)** | **Quercetin(n=35)** | **P value** |
| --- | --- | --- | --- |
| **FSH, IU/L** | **5.95+1.32** | **5.75+1.67** | **P=0.579** |
| **LH, IU/L** | **4.75±1.95** | **5.42±2.24** | **P=0.197** |
| **E2, pg/ml** | **35.11±13.84** | **35.37±18.07** | **P=0.948** |
| **T, ng/dl** | **23.57±12.98** | **24.96±10.93** | **P=0.633** |
| **AMH, ng/ul** | **4.27±3.67** | **4.99±4.34** | **P=0.458** |
| **Age, Yr** | **31.21±4.99** | **31.03±4.52** | **P=0.874** |
| **BMI, kg/m2** | **24.05±3.87** | **24.53±3.97** | **P=0.615** |

Note: Data are expressed as means ± SD; Student's t-test.
