# Supplementary material for: Quercetin promotes in vitro maturation of oocytes from humans and aged mice
Source: Cell Death Dis. 2020 Nov 11;11(11):965. doi: 10.1038/s41419-020-03183-5 (PMC7658351; doi:10.1038/s41419-020-03183-5)
Supplement: Supplementary file 5 — Antibody information [file 41419_2020_3183_MOESM5_ESM.docx]

**Table S2. Antibody information**

| **Protein name** | **Manufacture (catalogue number)** | **Applications(working dilution)** | **Uses** |
| --- | --- | --- | --- |
| LC3 | Cell Signaling(4108) | 1:300 | Confocal Microscope |
| Caspase3 | Abcam(ab13847) | 1:100 |  |
| SOD2K68ac | Abcam(ab137037) | 1:300 |  |
| SOD2 | Abcam(ab68155) | 1:50 |  |
| Anti-a-tubulin-FITC antibody | Sigma(F2168) | 1:800 |  |
| Lens culinaris (LCA)-FITC | Vectorlabs(FL-1041-5) | 1:200 |  |
| Alexa Fluor(anti-rabbit) 488 | Cell Signaling(4412S) | 1:500 |  |
| Fluorescein labeled Lens Culinaris Agglutinin | Vectorlabs(FL-1041-5) | 1:200 |  |
| MitoTracker Red | Invitrogen(M7512) | 200 nM |  |
| In Situ Cell Death Detection Kit | Roche(11684795910) | 1:2 |  |
| carboxy-H2DCF diacetate | Beyotime(S0033) | 10 mM |  |
| MitoProbe™ JC-1 Assay | Invitrogen(M34152) | 2 μM |  |
| Mounting Medium with DAPI | Vector(H-1200) | 10ml |  |
| DAPI | Solarbio(C0060) | 1mg/ml |  |
| SIRT3 | Proteintech(10099-1-AP) | 1:2000 | Western Blot |
| β-action | Proteintech(66009-1-Ig ) | 1:2000 |  |
| IRDye 680RD Goat Anti-Rabbit | Li-Cor(926-68071) | 1:5000 |  |
| IRDye 800CW Goat Anti-Mouse | Li-Cor(926-32210) | 1:5000 |  |
