# Supplementary material for: Quercetin promotes in vitro maturation of oocytes from humans and aged mice
Source: Cell Death Dis. 2020 Nov 11;11(11):965. doi: 10.1038/s41419-020-03183-5 (PMC7658351; doi:10.1038/s41419-020-03183-5)
Supplement: Supplementary file 6 — Primer Sequences [file 41419_2020_3183_MOESM6_ESM.docx]

**Table S3. Primer Sequences**

| **Gene name** | **Primers Sequence** | **Uses** |
| --- | --- | --- |
| *Hmga2* | F: 5'-AAGGCAGCAAAAACAAGAGC-3' | qPCR |
|  | R: 5'-GCAGGCTTCTTCTGAACGAC-3' |  |
| *Ube2e3* | F: 5'-GACAGGCAAAGGTCGGATGAT-3' |  |
|  | R: 5'-5'-ATCTGCATCTGAACTGCCACT-3' |  |
| *Nbr1* | F: 5'-GCAAGGAAATCAGCTACAGATGC-3' |  |
|  | R: 5'-ATCCCAAGACTCTCACCAGTG-3' |  |
| *Mtch2* | F: 5'-TGGGCGACAAGTATGTCAGC-3' |  |
|  | R: 5'-AGGACTCCTGAACACAGTCTT-3' |  |
| *Il6* | F: 5'-CTGCAAGAGACTTCCATCCAG-3' |  |
|  | R: 5'-AGTGGTATAGACAGGTCTGTTGG-3' |  |
| *CASP9* | F: 5'-GGCTGTTAAACCCCTAGACCA-3' |  |
|  | R: 5'-TGACGGGTCCAGCTTCACTA-3' |  |
| *sirt3* | F: 5'-TGGGGAGTGGTGCTTTTTATG-3' |  |
|  | R: GGGCAATGTAGGGTCGTCAG-3' |  |
| *Sod2* | F: 5'-AGGTCGGTGTGAACGGATTTG-3' |  |
|  | R: TGTAGACCATGTAGTTGAGGTCA-3' |  |
| *Gpx4* | F: 5'-ATCCCTTCGCGCCTGTACT-3' |  |
|  | R: GTGCTCGTAGATCGCGTCT-3' |  |
| *Bmp15* | F: 5'-GTCACCTCTACAATACCGTCCG-3' |  |
|  | R: CACCCGGTCCAGGTTAAACA-3' |  |
| *Gdf9* | F: 5'-GACCTGCCTTACGACTATG-3' |  |
|  | R: GAAGAGCGACCTGAGTTG-3' |  |
| *Gapdh* | F: 5'-AAATCAAGGAGTTTGCAGCCGG-3' |  |
|  | R: TTCTCTATCACCTGGGGCTCCT-3' |  |
| *Sirt3* | F: 5'-GCCTCTACAGCAACCTTCA-3' | siRNA |
